# Unleashing the Power of Contrastive Learning for Zero-Shot Video Summarization †

**DOI:** 10.3390/jimaging10090229

**Published:** 2024-09-14

**Authors:** Zongshang Pang, Yuta Nakashima, Mayu Otani, Hajime Nagahara

**Affiliations:** 1 Intelligence and Sensing Lab, Osaka University, Suita 565-0871, Japan; n-yuta@ids.osaka-u.ac.jp (Y.N.); nagahara@ids.osaka-u.ac.jp (H.N.); 2 CyberAgent, Inc., Tokyo 150-0042, Japan; otani_mayu@cyberagent.co.jp

**Keywords:** video summarization, contrastive learning, visual pre-training

## Abstract

Video summarization aims to select the most informative subset of frames in a video to facilitate efficient video browsing. Past efforts have invariantly involved training summarization models with annotated summaries or heuristic objectives. In this work, we reveal that features pre-trained on image-level tasks contain rich semantic information that can be readily leveraged to quantify frame-level importance for zero-shot video summarization. Leveraging pre-trained features and contrastive learning, we propose three metrics featuring a desirable keyframe: local dissimilarity, global consistency, and uniqueness. We show that the metrics can well-capture the diversity and representativeness of frames commonly used for the unsupervised generation of video summaries, demonstrating competitive or better performance compared to past methods when no training is needed. We further propose a contrastive learning-based pre-training strategy on unlabeled videos to enhance the quality of the proposed metrics and, thus, improve the evaluated performance on the public benchmarks TVSum and SumMe.

## 1. Introduction

In an era where video data are booming at an unprecedented pace, the importance of making the video browsing process more efficient has never been greater. Video summarization facilitates efficient browsing by creating a concise synopsis of the raw video, a topic that has been popular in research for many years. The rapid development of deep learning has significantly promoted the efficacy of video summarization tools [1]. Supervised approaches [2,3,4,5] leverage the temporal modeling power of LSTM (long short-term memory) [6] or self-attention mechanisms [7] and train them with annotated summaries. Heuristic training objectives such as diversity and representativeness have been applied using unsupervised methods [8,9,10,11,12,13,14] to enforce a diverse selection of keyframes that are representative of the essential contents of videos.

Past unsupervised approaches have trained summarization models to produce diverse and representative summaries by optimizing feature similarity-based loss/reward functions. Many research works on visual representation learning have revealed that vision models pre-trained on supervised or self-supervised tasks contain rich semantic signals, facilitating zero-shot transfer learning in tasks such as classification [15,16], semantic segmentation [17], and object detection [18]. In this work, we propose leveraging the rich semantics encoded in pre-trained visual features to achieve zero-shot video summarization that outperforms previous heavily-trained approaches and self-supervised pre-training to enhance the zero-shot performance further.

Specifically, we first define *local dissimilarity* and *global consistency* as two desirable criteria for localizing keyframe candidates. Inspired by the diversity objective, if a frame is distant from its nearest neighbors in the feature space, it encodes information that rarely appears in other frames. As a result, including such frames in the summary contributes to the diversity of its content. Such frames are considered to be decent key frame candidates as they enjoy high local dissimilarity, the naming of which leverages the definition of locality in the feature space in [19]. However, merely selecting frames based on dissimilarity may wrongly incorporate noisy frames that are not indicative of the video storyline. Therefore, we constrain the keyframes to be aligned with the video storyline by guaranteeing their high semantic similarity with the global cluster of the video frames, i.e., they are representative of (or globally consistent with) the video theme. Overall, the selected keyframes should enjoy a decent level of local dissimilarity to increase the content diversity in the summary and reflect the global video gist.

In contrast to previous works that required training to enforce the designed criteria, we directly quantify the proposed criteria into frame-level importance scores by utilizing contrastive losses for visual representation learning, i.e., alignment and uniformity losses [20]. The alignment loss calculates the distance between semantically similar samples, such as augmented versions of an input image, and minimizes this distance to ensure similarity between these positive samples in a contrastive learning setting. In our case, we directly apply the alignment loss to quantify the local dissimilarity metric. Uniformity loss is employed to regularize the overall distribution of features, with higher values indicating closely clustered features. This characteristic makes it well-suited for assessing the semantic consistency across a group of frames. To leverage this, we adapt the uniformity loss to evaluate the consistency between an individual frame and the entire set of video frames, which serves as a proxy for the global video storyline. These two losses can then be utilized for *self-supervised contrastive refinement* of the features, where contrastive learning is applied to optimize feature distances, ultimately enhancing the accuracy of the calculated frame importance scores.

**Figure 1:**
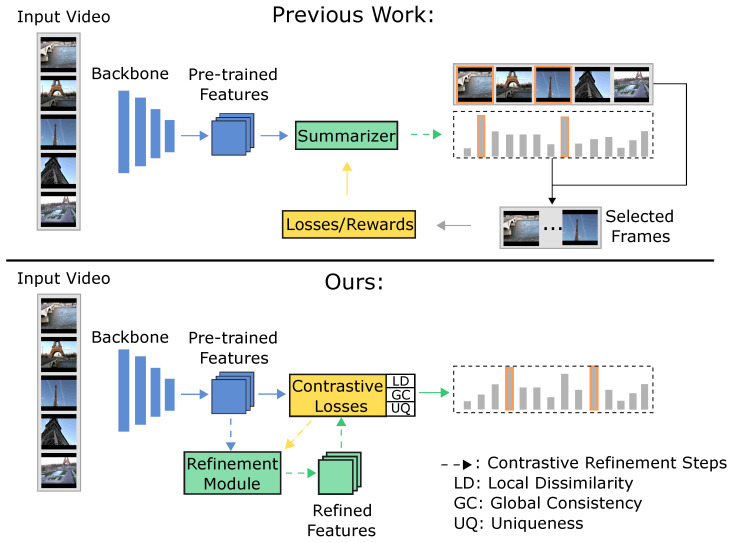
A comparison between our method and previous work.

Nonetheless, background frames may feature dynamic content that changes frequently, making them distinct from even the most similar frames and resulting in local dissimilarity. At the same time, these frames might contain background elements that are common across a majority of the video frames, contributing to global consistency. For example, in a video of a car accident, street scenes are likely to appear consistently. Although these frames might differ due to moving objects, they remain generally consistent with most frames, on average, due to the shared background context. We propose mitigating the chances of selecting such frames by exploiting the observation that such background frames tend to appear in many different videos with diverse topics and, thus, are not unique to their associated videos, e.g., street scenes in videos about car accidents, parades, city tours, etc. Specifically, we propose a *uniqueness filter* to quantify the uniqueness of frames, formulated by leveraging cross-video contrastive learning. An illustration of the difference between the proposed method and previous methods is provided in Figure 1.

Leveraging rich semantic information encoded in pre-trained visual features, we, for the first time, propose tackling training-free zero-shot video summarization and self-supervised pre-training to enhance the zero-shot transfer. Inspired by contrastive loss components [20], we achieve zero-shot summarization by quantifying frame importance into three metrics: local dissimilarity, global consistency, and uniqueness. The proposed method achieves better or competitive performance compared to previous methods while being training-free. Moreover, we introduce self-supervised contrastive refinement using unlabeled videos from YouTube-8M [21] to refine the feature distribution, which aids in training the proposed uniqueness filter and further enhances performance. Finally, compared to our conference paper [22], we provide results of current SOTA methods [23,24], provide more insightful analyses of the pros and cons of our proposed methods, and conduct more comprehensive ablation studies in various crucial aspects. The code to reproduce all the experiments is available at https://github.com/pangzss/pytorch-CTVSUM (accessed on 18 July 2024).

## 2. Related Work

Early applications in video summarization focus on sports videos [25,26,27] for event detection and highlight video compilation. Later on, video summarization was explored in other domains such as instructional videos [28,29,30,31], movies [32,33], and general user videos [34]. Thanks to the excellent generalization capabilities of deep neural networks/features, the focus of video summarization research has been diverted to developing general-purpose summarization models for a diverse range of video domains.

As an initial step toward deep learning-based supervised video summarization, Zhang et al. [2] utilized a long short-term memory (LSTM) for modeling temporal information when trained with human-annotated summaries, which sparked a series of subsequent works based on LSTM [3,35,36,37,38]. The rise of Transformer [7] inspired a suite of methods leveraging self-attention mechanisms for video summarization [4,5,10,39,40,41,42,43]. Some works have explored spatiotemporal information by jointly using RNNs and convolutional neural networks (CNNs) [44,45,46] or used graph convolution networks [47,48]. Video summarization leveraging multi-modal signals has also performed impressively [23,24,49].

Deep learning-based unsupervised methods mainly exploit two heuristics: diversity and representativeness. For diversity, some works [8,9,11,48] have utilized a diversity loss derived from a repelling regularizer [50], guaranteeing dissimilarities between selected keyframes. It has also been formulated as a reward function optimized via policy gradient methods, as seen in [12,51,52]. Similarly, representativeness can be guaranteed by reconstruction loss [8,10,11,13,53] or reconstruction-based reward functions [12,51,52].

Unlike previous works, we tackle training-free zero-shot video summarization and propose a pre-training strategy for better zero-shot transfer. Specifically, we directly calculate frame importance by leveraging contrastive loss terms formulated in [20] to quantify diversity and representativeness. With features from a vision backbone pre-trained on supervised image classification tasks [54] and without any further training, the proposed contrastive loss-based criteria can already well-capture the frame contribution to the diversity and representativeness of the summary. The proposed self-supervised contrastive refinement can further boost the performance and leverage unlabeled videos for zero-shot transfer to test videos.

## 3. Preliminaries

Given the centrality of contrastive learning to our approach, we first introduce the relevant preliminaries, with a focus on instance discrimination as outlined in [55].

### 3.1. Instance Discrimination via the InfoNCE Loss

Contrastive learning [56] has become a cornerstone of self-supervised image representation learning; throughout the years, it has received more attention from researchers. This method has been continuously refined to produce representations with exceptional transferability [19,20,53,55,57,58,59,60]. Formally, given a set of *N* images $D={\left\{I_{n}\right\}}_{n=1}^{N}$, contrastive representation learning aims to learn an encoder $f_{\theta }$ such that the resulting features $f_{\theta }\left(I_{n}\right)$ can be readily leveraged by downstream vision tasks. A theoretically founded loss function with favorable empirical behaviors is InfoNCE loss [58]:

(1) $$L_{\mathrm{InfoNCE}}=\sum_{I\in D}-\log\frac{e^{f_{\theta }\left(I\right)\cdot f_{\theta }\left(I^{\prime }\right)/\tau }}{\sum_{J\in D^{\prime }\left(I\right)}e^{f_{\theta }\left(I\right)\cdot f_{\theta }\left(J\right)/\tau }},$$

where $I^{\prime }$ is a positive sample for *I*, usually obtained through data augmentation, and $D^{\prime }\left(I\right)$ includes $I^{\prime }$ as well as all negative samples, e.g., any other images. The operator “·” is the inner product and τ is a temperature parameter. Therefore, the loss aims to pull the features of an instance closer to those of its augmented views while repelling them from the features of other instances, thus performing instance discrimination.

### 3.2. Contrastive Learning via Alignment and Uniformity

When normalized onto the unit hypersphere, the features learned through contrastive learning that yield strong downstream performance exhibit two notable properties. First, semantically related features tend to cluster closely on the sphere, regardless of specific details. Second, the overall information of the features is largely preserved, resulting in a joint distribution that approximates a uniform distribution [57,58,59]. Wang et al. [20] termed these two properties as *alignment* and *uniformity*.

The alignment metric computes the distance between the positive pairs [20]:

(2) $$L_{\mathrm{align}}\left(\theta ,\alpha \right)=\underset{\left(I,I^{\prime }\right)∼p_{\mathrm{pos}}}{E}[∥f_{\theta }\left(I\right)-f_{\theta }\left(I^{\prime }\right){∥}_{2}^{\alpha }],$$

where α>0, and $p_{\mathrm{pos}}$ is the distribution of positive pairs. The uniformity is defined as the average pairwise Gaussian potential between the overall features, as follows:

(3) $$L_{\mathrm{uniform}}\left(\theta ,\beta \right)=\log\left(\underset{I,J\overset{i.i.d}{∼}p_{\mathrm{data}}}{E}\left[e^{-\beta ∥f_{\theta }\left(I\right)-f_{\theta }{\left(J\right)∥}_{2}^{2}}\right]\right).$$

Here, $p_{\mathrm{data}}$ is typically approximated by the empirical data distribution, and β is commonly set to 2, as recommended by [20]. This metric promotes the overall feature distribution on the unit hypersphere to approximate a uniform distribution and can also directly quantify the uniformity of feature distributions [61]. Additionally, Equation (3) approximates the logarithm of the denominator in Equation (1) when the number of negative samples approaches infinity [20]. As demonstrated in [20], jointly minimizing Equations (2) and (3) leads to better alignment and uniformity of the features, meaning they become locally clustered and globally uniform [61].

In this paper, we employ Equation (2) to calculate the distance or dissimilarity between semantically similar video frame features, which helps measure frame importance based on local dissimilarity. We then apply a modified version of Equation (3) to assess the proximity between a specific frame and the overall information of the corresponding video, thereby estimating their semantic consistency. Additionally, by leveraging these two loss functions, we learn a nonlinear projection of the pre-trained features to enhance the local alignment and global uniformity of the projected features.

## 4. Proposed Method

**Figure 2:**
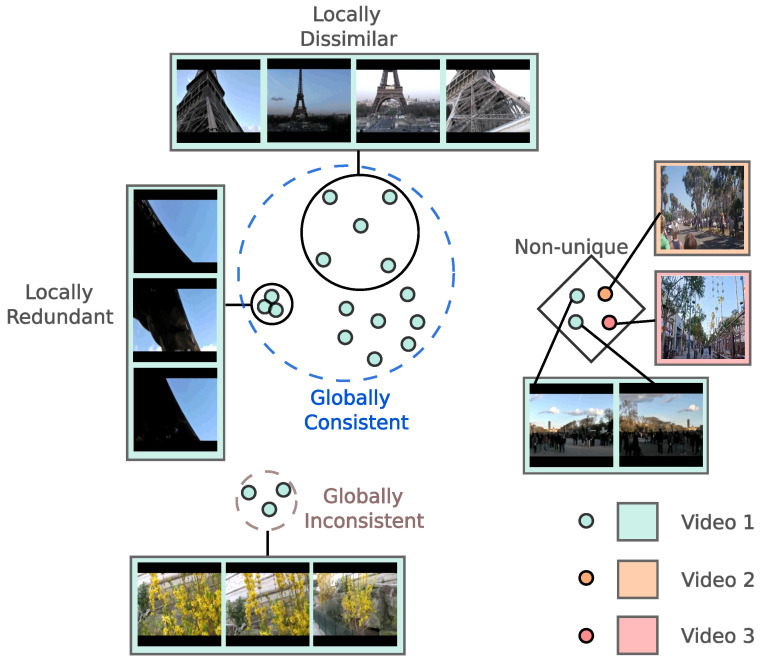
A conceptual illustration for the three metrics: local dissimilarity, global consistency, and uniqueness in the semantic space. The images come from the SumMe [34] and TVSum [31] datasets. The dots with the same color indicate features from the same video. For concise demonstration, we only show one frame for “Video 2” and “Video 3” to show the idea of uniqueness.

We first define two metrics to quantify frame importance by leveraging rich semantic information in pre-trained features: local dissimilarity and global consistency. To guarantee that the metrics encode the diversity and representativeness of the summary, we conduct self-supervised contrastive refinement of the features, where an extra metric called uniqueness is defined to further strengthen the keyframes’ quality. We provide a conceptual illustration of our approach in Figure 2.

### 4.1. Local Dissimilarity

Inspired by the diversity objective, we consider frames likely to result in a diverse summary as those conveying diverse information even when compared to their nearest neighbors. Formally, given a video V, we first extract deep features using the ImageNet [62] pre-trained vision backbone, e.g., GoogleNet [54], denoted as *F*, such that $F\left(V\right)={\left\{x_{t}\right\}}_{t=1}^{T}$, where $x_{t}$ represents the deep feature for the *t*-th frame in V, and *T* is the total number of frames in V. Each feature is L2-normalized such that ${∥x_{t}∥}_{2}=1$.

To define local dissimilarity for frames in V, we first use cosine similarity to retrieve for each frame $x_{t}$ a set $N_{t}$ of top K=aT neighbors, where *a* is a hyperparameter and *K* is rounded to the nearest integer. The local dissimilarity metric for $x_{t}$ is an empirical approximation of Equation (2), defined as the local alignment loss:

(4) $$L_{\mathrm{align}}\left(x_{t}\right)=\frac{1}{|N_{t}|}\sum_{x\in N_{t}}{∥x_{t}-x∥}_{2}^{2},$$

which measures the distance/dissimilarity between $x_{t}$ and its semantic neighbors.

The larger the value of $L_{\mathrm{align}}\left(x_{t}\right)$, the more dissimilar $x_{t}$ is from its neighbors. Therefore, if a frame exhibits a certain distance from even its closest neighbors in the semantic space, the frames within its local neighborhood are likely to contain diverse information, making them strong candidates for keyframes. Consequently, $L_{\mathrm{align}}\left(x_{t}\right)$ can be directly utilized as the importance score for $x_{t}$ after appropriate scaling.

### 4.2. Global Consistency

$N_{t}$ may contain semantically irrelevant frames if $x_{t}$ has very few meaningful semantic neighbors in the video. Therefore, merely using Equation (4) for frame-wise importance scores is insufficient. Inspired by the reconstruction-based representativeness objective [8], we define another metric, called global consistency, to quantify how consistent a frame is with the video gist by a modified uniformity loss based on Equation (3):

(5) $$L_{\mathrm{uniform}}\left(x_{t}\right)=\log\left(\frac{1}{T-1}\sum_{\begin{array}{c} x\neq x_{t},\\ x\in F(V) \end{array}}e^{-2∥x_{t}{-x∥}_{2}^{2}}\right),$$

$L_{\mathrm{uniform}}\left(x_{t}\right)$ measures the proximity between $x_{t}$ and the remaining frames, bearing similarity to the reconstruction- and K-medoid-based objectives in [8,12]. However, it obviates the need to train an autoencoder [8] or a policy network [12] by directly leveraging rich semantics in pre-trained features.

### 4.3. Contrastive Refinement

Equations (4) and (5) are computed using deep features pre-trained on image classification tasks, which may not inherently exhibit the local alignment and global uniformity described in Section 3.2. To address similar challenges, Hamilton et al. [17] proposed contrastively refining self-supervised vision transformer features [15] for unsupervised semantic segmentation. They achieve this by freezing the feature extractor (to improve efficiency) and training only a lightweight projector. Following this approach, we also avoid fine-tuning the heavy feature extractor—in our case, GoogleNet—and instead train only a lightweight projection head.

Formally, given features F(V) from the frozen backbone for a video, we feed them to a learnable module to obtain $z_{t}=G_{\theta }\left(x_{t}\right)$, where $z_{t}$ is L2-normalized (we leave out the L2-normalization operator for notation simplicity). The nearest neighbors in $N_{t}$ for each frame are still determined using the pre-trained features ${\left\{x_{t}\right\}}_{t=1}^{T}$. Similar to [19,63], we also observe collapsed training when directly using the learnable features for nearest neighbor retrieval, so we stick to using the frozen features.

With the learnable features, the alignment loss (local dissimilarity) and uniformity loss (global consistency) become (we slightly abuse the notation of L to represent losses both before and after transformation by $G_{\theta }$):

(6) $$\begin{array}{cc} L_{\mathrm{align}}\left(z_{t};\theta \right) & =\frac{1}{|N_{t}|}\sum_{z\in N_{t}}{∥z_{t}-z∥}_{2}^{2}, \end{array}$$

(7) $$\begin{array}{cc} L_{\mathrm{uniform}}\left(z_{t};\theta \right) & =\log\left(\frac{1}{T-1}\sum_{\begin{array}{c} z\neq z_{t},\\ z\in G_{\theta }\left(F\left(V\right)\right) \end{array}}e^{-2∥z_{t}{-z∥}_{2}^{2}}\right), \end{array}$$

The joint loss function is as follows:

(8) $$L\left(z_{t};\theta \right)=L_{\mathrm{align}}\left(z_{t};\theta \right)+\lambda_{1}L_{\mathrm{uniform}}\left(z_{t};\theta \right),$$

where $\lambda_{1}$ is a hyperparameter balancing the two loss terms.

During the contrastive refinement, $L_{\mathrm{align}}$ and $L_{\mathrm{uniform}}$ will mutually resist each other for frames that have semantically meaningful nearest neighbors and are consistent with the video gist. Specifically, when a nontrivial number of frames beyond $N_{t}$ also share similar semantic information with the anchor $z_{t}$, these frames function as “hard negatives” that prevent $L_{\mathrm{align}}$ to be easily minimized [19,61]. Therefore, only frames with moderate local dissimilarity and global consistency will have balanced values for the two losses. In contrast, the other frames tend to have extreme values compared to those before the refinement.

### 4.4. The Uniqueness Filter

The two metrics defined above fail to account for the fact that locally dissimilar yet globally consistent frames can often be background frames with complex content that is related to most of the frames in the video. For example, dynamic cityscapes might frequently appear in videos recorded in urban settings.

To address this, we propose filtering out such frames by leveraging a common characteristic: they tend to appear in many different videos that do not necessarily share a common theme or context. For instance, city views might be present in videos about car accidents, city tours, or parades, while scenes featuring people moving around can appear across various contexts. Consequently, these frames are not unique to their respective videos. This concept has been similarly explored in weakly-supervised action localization research [64,65,66], where a single class prototype vector is used to capture all background frames. However, our approach aims to identify background frames in an unsupervised manner. Additionally, rather than relying on a single prototype, which can be too restrictive [67], we treat each frame as a potential background prototype. By identifying frames that are highly activated across random videos, we develop a metric to determine the “background-ness” of a frame.

To design a filter for eliminating such frames, we introduce an extra loss to Equation (8) that taps into cross-video samples. For computational efficiency, we aggregate the frame features in a video $V_{k}$ with $T_{k}$ frames into segments with an equal length of *m*. The learnable features, z, in each segment, are average-pooled and L2-normalized to obtain segment features $S_{k}={\left\{s_{l}\right\}}_{l=1}^{|S_{k}|}$ with $|S_{k}|=\left\lfloor T_{k}/m\right\rfloor$. To measure the proximity of a frame with frames from a randomly sampled batch of videos B (represented as segment features), including $S_{k}$, we again leverage Equation (3) to define the uniqueness loss for $z_{t}\in V_{k}$ as follows:

(9) $$L_{\mathrm{unique}}\left(z_{t};\theta \right)=\log\left(\frac{1}{A}\sum_{S\in B/S_{k}}\sum_{s\in S}e^{-2∥z_{t}{-s∥}_{2}^{2}}\right),$$

where $A=\sum_{S\in B/S_{k}}\left|S\right|$ is the normalization factor. A large value of $L_{\mathrm{unique}}$ means that $z_{t}$ has nontrivial similarity with segments from randomly gathered videos, indicating that it is likely to be a background frame. When jointly optimized with Equations (8) and (9) the process will be easy to minimize for unique frames, for which most elements of s are semantically irrelevant and can be safely repelled. It is not the case for the background frames with semantically similar s, as the local alignment loss keeps strengthening the closeness of semantically similar features.

As computing Equation (9) requires random videos, it is not as straightforward to convert Equation (9) to importance scores after training. To address this, we train a model $H_{\hat{\theta }}$ whose last layer is a sigmoid unit to mimic $1-{\bar{L}}_{\mathrm{unique}}\left(z_{t};\theta \right)$, where ${\bar{L}}_{\mathrm{unique}}\left(z_{t};\theta \right)$ is $L_{\mathrm{unique}}\left(z_{t};\theta \right)$ scaled to [0,1] over *t*. Denoting $y_{t}=1-\mathrm{sg}\left({\bar{L}}_{\mathrm{unique}}\left(z_{t};\theta \right)\right)$ and $r_{t}=H_{\hat{\theta }}\left(\mathrm{sg}\left(z_{t}\right)\right)$, where “sg” stands for stop gradients, we define the loss for training the model as follows:

(10) $$L_{\mathrm{filter}}\left(z_{t};\hat{\theta }\right)=-y_{t}\log r_{t}+\left(1-y_{t}\right)\log\left(1-r_{t}\right).$$

### 4.5. The Full Loss and Importance Scores

With all the components, the loss for each frame in a video is as follows:

(11) $$\begin{array}{c} L\left(z_{t};\theta ,\hat{\theta }\right)=L_{\mathrm{align}}\left(z_{t};\theta \right)+\lambda_{1}L_{\mathrm{uniform}}\left(z_{t};\theta \right)\\ +\lambda_{2}L_{\mathrm{unique}}\left(z_{t};\theta \right)+\lambda_{3}L_{\mathrm{filter}}\left(z_{t};\hat{\theta }\right), \end{array}$$

where we fix both $\lambda_{2}$ and $\lambda_{3}$ as 0.1 and only tune $\lambda_{1}$.

Scaling the local dissimilarity, global consistency, and uniqueness scores to [0,1] over *t*, the frame-level importance score is defined as follows:

(12) $$p_{t}={\bar{L}}_{\mathrm{align}}\left(z_{t};\theta \right){\bar{L}}_{\mathrm{uniform}}\left(z_{t};\theta \right){\bar{H}}_{\hat{\theta }}\left(z_{t}\right)+\epsilon ,$$

which ensures that the importance scores are high only when all three terms have significant magnitude. The parameter ϵ is included to prevent zero values in the importance scores, which helps stabilize the knapsack algorithm used to generate the final summaries. Since these scores are derived from three independent metrics, they may lack the temporal smoothness typically provided by methods like RNNs [2] or attention networks [5]. To address this, we apply Gaussian smoothing to the scores within each video, aligning our method with previous work that emphasizes the importance of temporal smoothness in score generation.

## 5. Experiments

### 5.1. Datasets and Settings

**Datasets.** In line with previous studies, we evaluate our method on two benchmarks: TVSum [31] and SumMe [34]. TVSum consists of 50 YouTube videos, each annotated by 20 individuals who provide importance scores for every two-second shot. SumMe includes 25 videos, each with 15 to 18 reference binary summaries. Following the protocol established by [2], we use the OVP (50 videos) and YouTube (39 videos) datasets [68] to augment both TVSum and SumMe. Additionally, to assess whether our self-supervised approach can benefit from a larger video dataset, we randomly selected approximately 10,000 videos from the YouTube-8M dataset [21], which contains 3862 video classes with highly diverse content.

**Evaluation Setting.** Following prior work, we evaluate our model’s performance using five-fold cross-validation, where the dataset (either TVSum or SumMe) is randomly divided into five splits. The reported results are the average across these five splits. In the canonical setting (C), training is performed only on the original splits of the two evaluation datasets. In the augmented setting (A), we expand the training set in each fold with three additional datasets (e.g., SumMe, YouTube, and OVP when evaluating on TVSum). In the transfer setting (T), all videos from TVSum (or SumMe) are reserved for testing, while the other three datasets are used for training. Additionally, we introduce a new transfer setting where training is exclusively conducted on the collected YouTube-8M videos, and evaluation is performed on TVSum or SumMe. This setting is intended to assess whether our model can benefit from a larger volume of data.

### 5.2. Evaluation Metrics

**F1 score**. Denoting *A* as the set of frames in a ground-truth summary and *B* as the set of frames in the corresponding generated summary, we can calculate precision and recall as follows:

(13) $$\mathrm{Precision}=\frac{\left|A\cap B\right|}{\left|A\right|},\;\mathrm{Recall}=\frac{\left|A\cap B\right|}{\left|B\right|},$$

with which we can calculate the F1 score by the following:

(14) $$F1=\frac{2\times \mathrm{Precision}\times \mathrm{Recall}}{\mathrm{Precision}+\mathrm{Recall}}.$$

We follow [2] to deal with multiple ground-truth summaries and to convert importance scores into summaries.

**Rank correlation coefficients.** Recently, Otani et al. [69] highlighted that F1 scores can be unreliable and may yield relatively high values even for randomly generated summaries. To address this issue, they proposed using rank correlation coefficients, specifically Kendall’s τ [70] and Spearman’s ρ [71], to evaluate the correlation between predicted and ground-truth importance scores. For each video, we first compute the coefficient value between the predicted importance scores and the scores provided by each annotator, then average these values across all annotators for that video. The final results are obtained by averaging the correlation coefficients across all videos.

### 5.3. Summary Generation

We follow previous work to convert importance scores to key shots. Specifically, we use the KTS algorithm [72] to segment videos into temporally consecutive shots and then average the importance scores within each shot to compute the shot-level importance scores. The final key shots are chosen to maximize the total score while guaranteeing that the summary length does not surpass 15% of the video length. The maximization is conducted by solving the knapsack problem based on dynamic programming [31]. Otani et al. [69] pointed out that using average frame importance scores as shot-level scores will drastically increase the F1 score for the TVSum dataset, and they recommended using the sum of scores to alleviate the problem. However, F1 scores reported by previous works mostly rely on averaging importance scores for shot-level scores. We also report our F1 scores in the same way as they did but focus on analyzing the rank correlation values for comparison and analysis.

### 5.4. Implementation Details

We follow prior studies by using GoogleNet [54] pre-trained features as the default for standard experiments. For experiments involving YouTube-8M videos, we utilize the quantized Inception-V3 [73] features provided by the dataset [21]. Both types of features are pre-trained on ImageNet [62]. The contrastive refinement module appended to the feature backbone is a lightweight Transformer encoder [7], and so is the uniqueness filter.

Following [9], we standardized each video to have an equal length by using random sub-sampling for longer videos and nearest-neighbor interpolation for shorter videos. Similar to [9], we did not observe much difference when using different lengths, and we fixed the frame count at 200.

The model appended to the feature backbone for contrastive refinement is a stack of Transformer encoders with multi-head attention modules [7]. There are two training scenarios: 1. Training with TVSum [31], SumMe [34], YouTube, and OVP [68], divided into the canonical, augmented, and transfer settings; 2. Training with a subset of videos from the YouTube-8M dataset [21]. We refer to the training in the first scenario as *standard* and the second as *YT8M*. The pre-trained features are first projected into 128 dimensions for training in both scenarios using a learnable, fully connected layer. The projected features are then fed into the Transformer encoders. The model architecture and associated optimization details are outlined in Table 1. Training the 10,000 YouTube-8M videos takes approximately 6 min for 40 epochs on a single NVIDIA RTX A6000.

We tune two hyperparameters: The ratio *a*, which determines the size of the nearest neighbor set $N_{t}$ and the coefficient $\lambda_{1}$, which controls the balance between the alignment and uniformity losses.

### 5.5. Quantitative Results

In this section, we compare our results with previous work and conduct the ablation study for different components of our method.

**Training-free zero-shot performance.** As shown in Table 2 and Table 3, ${\bar{L}}_{\mathrm{align}}^{*}$ and ${\bar{L}}_{\mathrm{uniform}}^{*}$ directly computed using GoogleNet [54] pre-trained features, achieve performance superior to most methods in terms of τ, ρ, and F1 score. Notably, the correlation coefficients τ and ρ surpass supervised methods, e.g., (0.1345, 0.1776) v.s. dppLSTM’s (0.0298, 0.0385) and SumGraph’s (0.094, 0.138) for TVSum. Although DR-DSN_2000_ has slightly better performance in terms of τ and ρ for TVSum, it has to reach the performance after 2000 epochs of training, while our results are directly obtained with simple computations using the same pre-trained features as those also used by DR-DSN.

**More training videos are needed for the contrastive refinement.** For the results in Table 2 and Table 3, the maximum number of training videos is only 159, coming from the SumMe augmented setting. For the canonical setting, the training set size is 40 videos for TVSum and 20 for SumMe. Without experiencing many videos, the model tends to overfit specific videos and cannot generalize well. This is similar to the observation in contrastive representation learning, where a larger amount of data—whether from a larger dataset or obtained through data augmentation—helps the model generalize better [15,60]. Therefore, the contrastive refinement results in Table 2 and Table 3 hardly outperform those computed using pre-trained features.

**Contrastive refinement on YouTube-8M videos and transfer to TVSum**. The model generalizes to the test videos better when sufficient training videos are given, as shown by the results for TVSum in Table 4. After the contrastive refinement, the results with only ${\bar{L}}_{\mathrm{align}}^{*}$ are improved from (0.0595, 0.0779) to (0.0911, 0.1196) for τ and ρ. We can also observe improvement over ${\bar{L}}_{\mathrm{align}}^{*}\;\&\;{\bar{L}}_{\mathrm{uniform}}^{*}$ brought by contrastive refinement.

**Contrastive refinement on YouTube-8M videos and transfer to SumMe**. The reference summaries in SumMe are binary scores, and summary lengths are constrained to be within 15% of the video lengths. Therefore, the majority of the reference summary receives exactly zero scores. The contrastive refinement may still enhance the confidence scores for these regions, which receive zero scores from annotators due to the 15% constraint. This can ultimately reduce the average correlation with the reference summaries, as seen in Table 4.

Suppose that the predicted scores are refined to have sufficiently high confidence for regions with nonzero annotated scores; in this case, they are likely to be selected by the knapsack algorithm used to compute the F1 scores. Therefore, we consider scores that achieve both high F1 and high correlations to be of high quality, as the former tends to overlook the overall correlations between the predicted and annotated scores [69], while the latter focuses on their overall ranked correlations but places less emphasis on prediction confidence. This analysis may explain why the contrastive refinement for ${\bar{L}}_{\mathrm{align}}^{*}$ improves the F1 score but decreases the correlations.

**The effect of ${\bar{L}}_{\mathrm{align}}$.** As can be observed in Table 2, Table 3 and Table 4, solely using ${\bar{L}}_{\mathrm{align}}$ can already well-quantify the frame importance. This indicates that ${\bar{L}}_{\mathrm{align}}$ successfully selects frames with diverse semantic information, which are indeed essential for a desirable summary. Moreover, we assume that diverse frames form the foundation of a good summary, consistently using ${\bar{L}}_{\mathrm{align}}$ for further ablations.

**The effect of ${\bar{L}}_{\mathrm{uniform}}$.**${\bar{L}}_{\mathrm{uniform}}$ measures how consistent a frame is with the context of the whole video, thus helping remove frames with diverse contents that are hardly related to the video theme. It is shown in Table 2 and Table 4 that incorporating ${\bar{L}}_{\mathrm{uniform}}$ helps improve the quality of the frame importance for TVSum. We now discuss why ${\bar{L}}_{\mathrm{uniform}}$ hurts SumMe performance.

**Figure 3:**
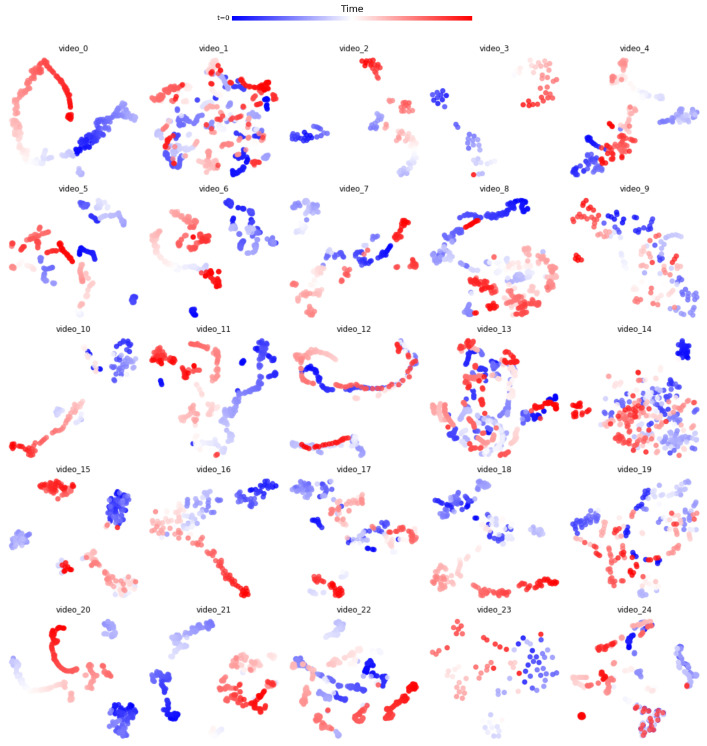
TSNE plots for all 25 SumMe videos. As can be observed, many videos contain features that slowly evolve and maintain an overall similarity among all the frames.

**Figure 4:**
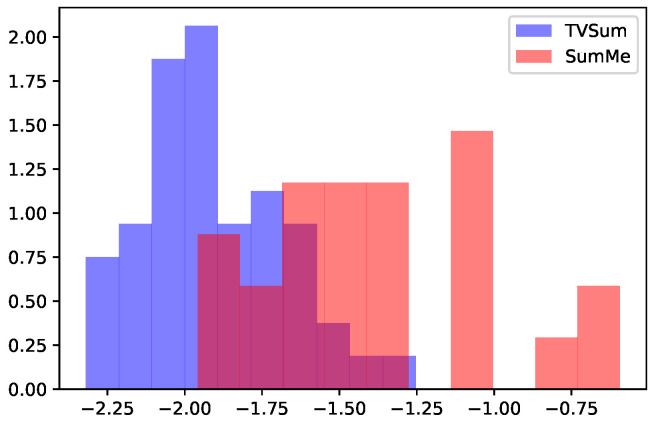
The histogram (density) of ${\bar{L}}_{\mathrm{uniform}}^{*}$ (before normalization) for TVSum and SumMe videos. SumMe videos have distinctly higher values than those for TVSum videos.

Compared to TVSum videos, many SumMe videos already contain consistent frames due to their slowly evolving properties. Such slowly evolving features can be visualized by T-SNE plots in Figure 3. For videos with such consistent content, the ${\bar{L}}_{\mathrm{uniform}}$ tends to be high for most of the frames. We show the normalized histogram of $L_{\mathrm{uniform}}^{*}$ for both TVSum and SumMe videos in Figure 4. As can be observed, SumMe videos have distinctly higher $L_{\mathrm{uniform}}^{*}$ than those of TVSum videos. Consequently, for videos possessing monotonous content, most of the frames share a similar visual cue, such as the background, and the frames that are most likely to be keyframes are those with abrupt or novel content. Therefore, the global consistency metric, ${\bar{L}}_{\mathrm{uniform}}^{*}$, is not discriminative enough to be sufficiently helpful and may alleviate the importance of frames with novel content. As a result, the other two metrics—local dissimilarity and uniqueness—are the main roles that determine keyframes in such videos, as shown in Table 2, Table 3 and Table 4.

**The effect of the uniqueness filter ${\bar{H}}_{\hat{\theta }}$.** As shown in Table 2 and Table 3, although ${\bar{H}}_{\hat{\theta }}$ works well for TVSum videos, it hardly brings any benefits to the SumMe videos. Thus, the good performance of the uniqueness filter for TVSum may be due to the relatively straightforward nature of the background frames in TVSum, which are easily identified by the uniqueness filter even with training on only a few videos. Therefore, we suppose that ${\bar{H}}_{\hat{\theta }}$ needs to be trained on more videos to filter out more challenging background frames such that it can generalize to a wider range of videos. This is validated by the ${\bar{L}}_{\mathrm{align}}$ & ${\bar{H}}_{\hat{\theta }}$ results in Table 4, which indicate both decent F1 scores and correlation coefficients for both TVSum and SumMe. The TVSum performance can be further boosted when ${\bar{L}}_{\mathrm{uniform}}$ is incorporated.

**Comparison with DR-DSN [12] on YouTube-8M.** As per Table 2, DR-DSN is the only unsupervised method that matches our performance in terms of τ and ρ and has an official implementation available. We trained DR-DSN on our dataset of YouTube-8M videos to compare it against our method. As shown in Table 4, DR-DSN has difficulty generalizing to the evaluation videos.

**Figure 5:**
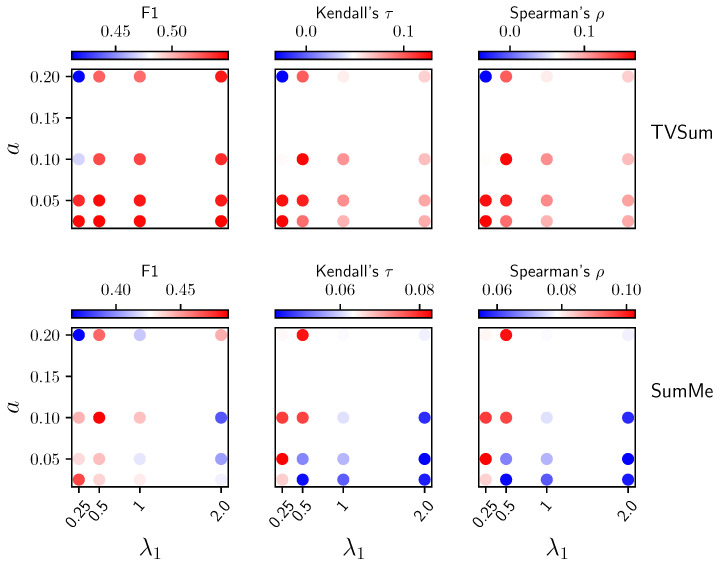
Ablation results over $\lambda_{1}$ and *a* when using ${\bar{L}}_{\mathrm{align}}$ & ${\bar{H}}_{\hat{\theta }}$ to produce importance scores.

**Figure 6:**
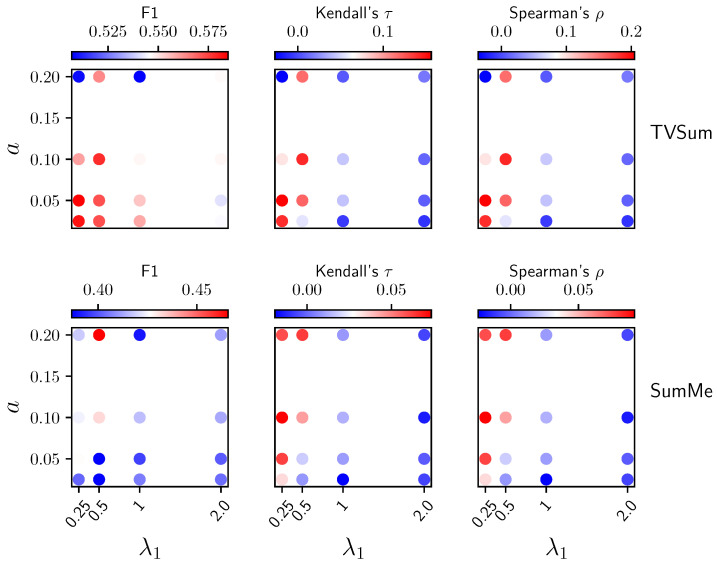
Ablation results over $\lambda_{1}$ and *a* when using ${\bar{L}}_{\mathrm{align}}$ & ${\bar{H}}_{\hat{\theta }}$ & ${\bar{L}}_{\mathrm{uniform}}$ to produce importance scores.

**Ablations over**$\lambda_{1}$**and***a***.** As shown in Figure 5, when ${\bar{L}}_{\mathrm{align}}$ & ${\bar{H}}_{\hat{\theta }}$ is used to produce importance scores, a larger *a* will make the TVSum performance unstable in terms of both F1 and correlation coefficients, although the SumMe performance is relatively more stable with respect to *a*. We hypothesize that when *a* becomes larger, the nearest neighbor set becomes noisier, diminishing the effectiveness of both the alignment loss during training and the local dissimilarity metric (post-training alignment loss) used for generating importance scores, due to the inclusion of semantically irrelevant neighbors. For $\lambda_{1}$, smaller values generally perform better when *a* has a reasonable value, as larger values of $\lambda_{1}$ tend to make the uniformity loss suppress the alignment loss. Similarly, too small $\lambda_{1}$ will make the alignment loss suppress the uniformity loss, as we observed unstable training when further decreasing $\lambda_{1}$. As shown in Figure 6, the analysis of the interaction between $\lambda_{1}$ and *a* when using ${\bar{L}}_{\mathrm{align}}$ & ${\bar{H}}_{\hat{\theta }}$ & ${\bar{L}}_{\mathrm{uniform}}$ is used to produce importance scores, similar to that in Figure 5. However, we can see that the performance was improved for TVSum but undermined for SumMe due to incorporating ${\bar{L}}_{\mathrm{uniform}}$.

**Ablation on model sizes.**Table 5 shows the ablation results for different sizes of the Transformer encoder [7], where the number of layers and the number of attention heads are varied. Meanwhile, we compare the results with those obtained from DR-DSN [12] trained on the same collected YouTube-8M videos, as DR-DSN has the best τ and ρ among past unsupervised methods and is the only one that has a publicly available official implementation. As can be observed, the model performance is generally stable with respect to the model sizes, and we choose 4L8H. Moreover, the DR-DSN has difficulty generalizing well to the test videos when trained on the YouTube-8M videos.

**Comparing the effects of different pre-trained features.** As our method can directly compute importance scores using pre-trained features, it is also essential for it to be able to work with different kinds of pre-trained features. To prove this, we computed and evaluated the importance scores generated with 2D supervised features, 3D supervised features, and 2D self-supervised features in Table 6. Different 2D features, whether supervised or self-supervised, all delivered decent results. Differences exist but are trivial. The conclusion that ${\bar{L}}_{\mathrm{unif}}$ helps TVSum but undermines SumMe also holds for most of the features. Based on this, we conclude that as long as the features contain decent semantic information learned from supervision or self-supervision, they are enough to efficiently compute the importance scores. The performance of these features transferred to different downstream image tasks does not necessarily generalize to our method for video summarization, as the latter only requires reliable semantic information (quantified as dot products) to calculate heuristic metrics for video frames.

Notably, our method does not perform optimally with 3D supervised video features. This outcome is anticipated because these 3D features are trained to encode information based on video-level labels, thus capturing less detailed semantic information in individual frames, which is crucial for our method. Still, such 3D features contain part of the holistic information of the associated video and may be a good vehicle for video summarization, which can benefit from such information.

### 5.6. Qualitative Results

**Figure 7:**
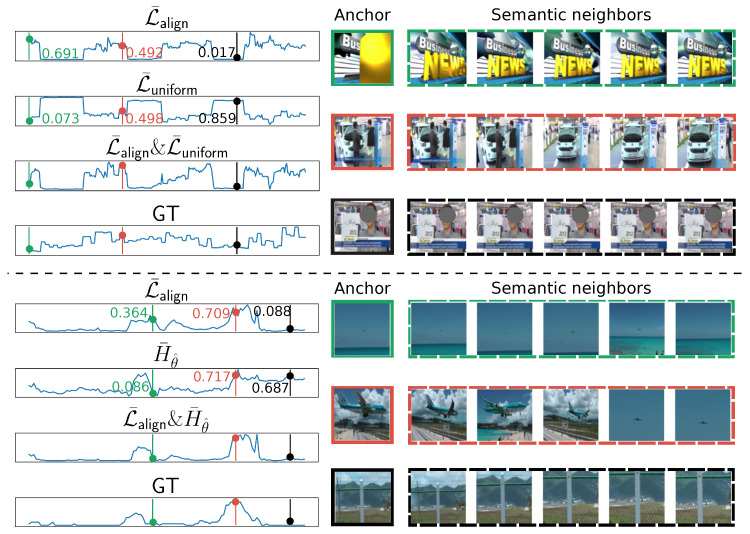
The qualitative analysis of two video examples. The left column contains importance scores, where “GT” stands for ground truth. The green bar selects an anchor frame with high ${\bar{L}}_{\mathrm{align}}$ but low ${\bar{L}}_{\mathrm{uniform}}$ or ${\bar{H}}_{\hat{\theta }}$, the red bar selects one with non-trial magnitude for both metrics, and the **black** bar selects one with low ${\bar{L}}_{\mathrm{align}}$ but high ${\bar{L}}_{\mathrm{uniform}}$ or ${\bar{H}}_{\hat{\theta }}$. We show five samples from the top 10 semantic nearest neighbors within the dashed boxes on the right for each selected anchor frame.

We show the effect of the local dissimilarity (${\bar{L}}_{\mathrm{align}}$), the global consistency (${\bar{L}}_{\mathrm{uniform}}$), and the uniqueness scores generated by the uniqueness filter ${\bar{H}}_{\hat{\theta }}$ in Figure 7. We visualize and discuss the effects in pairs, i.e., ${\bar{L}}_{\mathrm{align}}$ & ${\bar{L}}_{\mathrm{uniform}}$ and ${\bar{L}}_{\mathrm{align}}$ & ${\bar{H}}_{\hat{\theta }}$. In the upper half of Figure 7, the green bar selects a frame with high local similarity but low global consistency, which is a title frame with a disparate appearance and hardly conveys any valuable information about the video. While the **black** bar selects a frame related to the main content of the video (an interview), it has semantic neighbors with almost the same look and is less likely to contain diverse semantics. The red bar selects a frame with moderate local dissimilarity and global consistency. This frame, along with its semantic neighbors, conveys diverse information; for example, the car with or without people surrounding it. Moreover, it is highly relevant to the overall video context: an interview at a car company.

For the lower half of Figure 7, the green bar selects a frame with information noticeably different from its neighbors, e.g., the sea occupies different proportions of the scene. However, such a frame can appear in any video with water scenes, rendering it not unique to the belonging video. Hence, its uniqueness score is low. The **black** bar selects a frame with an object specifically belonging to this video in the center, but the local semantic neighborhood around it hardly conveys diverse information. The red bar selects a frame with both high local dissimilarity and high uniqueness, which is the frame related to the gist of the video: St. Maarten landing.

## 6. Conclusions

We make the first attempt to approach training-free, zero-shot video summarization by leveraging pre-trained deep features. We utilize contrastive learning to propose three metrics—local dissimilarity, global consistency, and uniqueness—to generate frame importance scores. The proposed metrics directly enable the creation of summaries with quality that is better or competitive compared to previous supervised or unsupervised methods requiring extensive training. Moreover, we propose contrastive pre-training on unlabeled videos to further boost the quality of the proposed metrics, the effectiveness of which has been verified by extensive experiments. It would be interesting to explore multi-modal zero-hot video summarization for future work.

## Figures and Tables

**Table 1:** Model and optimization details.

	Layers	Heads	$d_{\mathrm{model}}$	$d_{\mathrm{head}}$	$d_{\mathrm{inner}}$	Optimizer	LR	Weight Decay	Batch Size	Epoch	Dropout
Standard	4	1	128	64	512	Adam	0.0001	0.0001	32 (TVSum) 8 (SumMe)	40	0
YT8M	4	8	128	64	512	Adam	0.0001	0.0005	128	40	0

**Table 2:** Ablation results in terms of τ and ρ, along with their comparisons to previous work in the canonical setting. DR-DSN_60_ refers to the DR-DSN trained for 60 epochs; similarly, DR-DSN_2000_. Our scores with superscript ∗ are directly computed from pre-trained features. The results were generated with $\left(\lambda_{1},a\right)=\left(0.5,0.1\right)$. **Boldfaced** scores represent the best among supervised methods, and blue scores are the best among the methods without using annotations. Methods with † are vision–language approaches. Please refer to the text for analyses of the results.

	TVSum		SumMe	
	τ	ρ	τ	ρ
Human baseline [74]	0.1755	0.2019	0.1796	0.1863
*Supervised*				
VASNet [5,74]	0.1690	0.2221	0.0224	0.0255
dppLSTM [2,69]	0.0298	0.0385	−0.0256	−0.0311
SumGraph [48]	0.094	0.138	-	-
Multi-ranker [74]	**0.1758**	**0.2301**	0.0108	0.0137
Clip-It ^†^ [23]	0.108	0.147	-	
A2Summ^†^ [24]	0.137	0.165	**0.108**	**0.129**
*Unsupervised*				
DR-DSN_60_ [12,69]	0.0169	0.0227	0.0433	0.0501
DR-DSN_2000_ [12,74]	0.1516	0.198	−0.0159	−0.0218
SUM-FCN_unsup_ [9,74]	0.0107	0.0142	0.0080	0.0096
SUM-GAN [8,74]	−0.0535	−0.0701	−0.0095	−0.0122
CSNet + GL + RPE [14]	0.070	0.091	-	-
*Training-free*				
${\bar{L}}_{\mathrm{align}}^{*}$	0.1055	0.1389	0.0960	0.1173
${\bar{L}}_{\mathrm{align}}^{*}$ & ${\bar{L}}_{\mathrm{uniform}}^{*}$	0.1345	0.1776	0.0819	0.1001
*Contrastively refined*				
${\bar{L}}_{\mathrm{align}}$	0.1002	0.1321	0.0942	0.1151
${\bar{L}}_{\mathrm{align}}$ & ${\bar{L}}_{\mathrm{uniform}}$	0.1231	0.1625	0.0689	0.0842
${\bar{L}}_{\mathrm{align}}$ & ${\bar{H}}_{\hat{\theta }}$	0.1388	0.1827	0.0585	0.0715
${\bar{L}}_{\mathrm{align}}$ & ${\bar{L}}_{\mathrm{uniform}}$ & ${\bar{H}}_{\hat{\theta }}$	0.1609	0.2118	0.0358	0.0437

**Table 3:** Ablation results regarding F1 and their comparisons with previous unsupervised methods. The **boldfaced** results are the best ones. Please refer to Table 2’s caption for the explanation of the notations and the text for analyses of the results.

	TVSum			SumMe		
	C	A	T	C	A	T
*Unsupervised*						
DR-DSN_60_ [12]	57.6	58.4	57.8	41.4	42.8	42.4
SUM-FCN_unsup_ [9]	52.7	-	-	41.5	-	39.5
SUM-GAN [8]	51.7	59.5	-	39.1	43.4	-
UnpairedVSN [11]	55.6	-	55.7	47.5	-	41.6
CSNet [13]	58.8	59	59.2	**51.3**	**52.1**	45.1
CSNet + GL + RPE [14]	59.1	-	-	50.2	-	-
SumGraph_unsup_ [48]	59.3	**61.2**	57.6	49.8	**52.1**	**47**
*Training-free*						
${\bar{L}}_{\mathrm{align}}^{*}$	56.4	56.4	54.6	43.5	43.5	39.4
${\bar{L}}_{\mathrm{align}}^{*}$ & ${\bar{L}}_{\mathrm{uniform}}^{*}$	58.4	58.4	56.8	47.2	46.07	41.7
*Contrastively refined*						
${\bar{L}}_{\mathrm{align}}$	54.6	55.1	53	46.8	47.1	41.5
${\bar{L}}_{\mathrm{align}}$ & ${\bar{L}}_{\mathrm{uniform}}$	58.8	59.9	57.4	46.7	48.4	41.1
${\bar{L}}_{\mathrm{align}}$ & ${\bar{H}}_{\hat{\theta }}$	53.8	56	54.3	45.2	45	45.3
${\bar{L}}_{\mathrm{align}}$ & ${\bar{L}}_{\mathrm{uniform}}$ & ${\bar{H}}_{\hat{\theta }}$	**59.5**	59.9	**59.7**	46.8	45.5	43.9

**Table 4:** The transfer evaluation setting with the YouTube-8M dataset, where the training is solely conducted on the collected YouTube-8M videos and then evaluated on TVSum and SumMe. The results from DR-DSN [12] are also provided for comparison.

	TVSum			SumMe		
	F1	τ	ρ	F1	τ	ρ
*Unsupervised*						
DR-DSN [12]	51.6	0.0594	0.0788	39.8	−0.0142	−0.0176
*Training-free*						
${\bar{L}}_{\mathrm{align}}^{*}$	55.9	0.0595	0.0779	45.5	0.1000	0.1237
${\bar{L}}_{\mathrm{align}}^{*}$ & ${\bar{L}}_{\mathrm{uniform}}^{*}$	56.7	0.0680	0.0899	42.9	0.0531	0.0649
*Contrastively refined*						
${\bar{L}}_{\mathrm{align}}$	56.2	0.0911	0.1196	46.6	0.0776	0.0960
${\bar{L}}_{\mathrm{align}}$ & ${\bar{L}}_{\mathrm{uniform}}$	57.3	0.1130	0.1490	40.9	0.0153	0.0190
${\bar{L}}_{\mathrm{align}}$ & ${\bar{H}}_{\hat{\theta }}$	58.1	0.1230	0.1612	48.7	0.0780	0.0964
${\bar{L}}_{\mathrm{align}}$ & ${\bar{L}}_{\mathrm{uniform}}$ & ${\bar{H}}_{\hat{\theta }}$	59.4	0.1563	0.2048	43.2	0.0449	0.0553

**Table 5:** Ablation results for the model size and comparison with DR-DSN [12] trained on the same YouTube-8M videos, where 2L2H represents “2 layers 2 heads” and the rest goes similarly. All three components ${\bar{L}}_{\mathrm{align}}$ & ${\bar{H}}_{\hat{\theta }}$ & ${\bar{L}}_{\mathrm{uniform}}$ are used with $\left(a,\lambda_{1}\right)=\left(0.05,0.25\right)$ for both SumMe and TVSum for fair comparison with DR-DSN, which also uses a representativeness-based training objective.

	TVSum			SumMe		
	F1	τ	ρ	F1	τ	ρ
DR-DSN [12]	51.6	0.0594	0.0788	39.8	−0.0142	−0.0176
2L2H	58.0	0.1492	0.1953	42.9	0.0689	0.0850
2L4H	58.1	0.1445	0.1894	42.8	0.0644	0.0794
2L8H	58.8	0.1535	0.2011	44.0	0.0584	0.0722
4L2H	57.4	0.1498	0.1963	45.3	0.0627	0.0776
4L4H	58.3	0.1534	0.2009	43.1	0.0640	0.0790
4L8H	58.5	0.1564	0.2050	42.7	0.0618	0.0765

**Table 6:** Evaluation results with different pre-trained features. The results were produced under the transfer setting with a=0.1.

	TVSum						SumMe					
	${\bar{L}}_{\mathrm{align}}^{*}$			${\bar{L}}_{\mathrm{align}}^{*}$ & ${\bar{L}}_{\mathrm{unif}}^{*}$			${\bar{L}}_{\mathrm{align}}^{*}$			${\bar{L}}_{\mathrm{align}}^{*}$ & ${\bar{L}}_{\mathrm{unif}}^{*}$		
	F1	τ	ρ	F1	τ	ρ	F1	τ	ρ	F1	τ	ρ
*Supervised (2D)*												
VGG19 [75]	50.62	0.0745	0.0971	55.91	0.1119	0.1473	45.16	0.0929	0.1151	43.28	0.0899	0.1114
GoogleNet [54]	54.67	0.0985	0.1285	57.09	0.1296	0.1699	41.89	0.0832	0.1031	40.97	0.0750	0.0929
InceptionV3 [73]	55.02	0.1093	0.1434	55.63	0.0819	0.1082	42.71	0.0878	0.1087	42.30	0.0688	0.0851
ResNet50 [76]	51.19	0.0806	0.1051	55.19	0.1073	0.1410	42.30	0.0868	0.1076	43.86	0.0737	0.0914
ResNet101 [76]	51.75	0.0829	0.1081	54.88	0.1118	0.1469	42.32	0.0911	0.1130	44.39	0.0736	0.0913
ViT-S-16 [77]	53.48	0.0691	0.0903	56.15	0.1017	0.1332	40.30	0.0652	0.0808	40.88	0.0566	0.0701
ViT-B-16 [77]	52.85	0.0670	0.0873	56.15	0.0876	0.1152	42.10	0.0694	0.0860	41.65	0.0582	0.0723
Swin-S [78]	52.05	0.0825	0.1082	57.58	0.1120	0.1475	41.18	0.0880	0.1090	41.63	0.0825	0.1022
*Supervised (3D)*												
R3D50 [79]	52.09	0.0590	0.0766	53.35	0.0667	0.0869	37.40	0.0107	0.0138	41.03	0.0150	0.0190
R3D101 [79]	49.77	0.0561	0.0727	52.15	0.0644	0.0834	33.62	0.0173	0.0216	34.96	0.0212	0.0264
*Self-supervised (2D)*												
MoCo [80]	51.31	0.0797	0.1034	55.97	0.1062	0.1390	42.01	0.0768	0.0953	43.19	0.0711	0.0882
DINO-S-16 [15]	52.50	0.0970	0.1268	57.57	0.1200	0.1583	42.77	0.0848	0.1050	42.67	0.0737	0.0913
DINO-B-16 [15]	52.48	0.0893	0.1170	57.02	0.1147	0.1515	41.07	0.0861	0.1066	44.14	0.0679	0.0843
BEiT-B-16 [81]	49.64	0.1125	0.1468	56.34	0.1270	0.1665	36.91	0.0554	0.0686	38.48	0.0507	0.0629
MAE-B-16 [82]	50.40	0.0686	0.0892	54.58	0.1013	0.1327	40.32	0.0560	0.0695	39.46	0.0484	0.0601

## Data Availability

TVSum: https://github.com/yalesong/tvsum, accessed on 18 July 2024. SumMe: https://paperswithcode.com/dataset/summe, accessed on 18 July 2024. YouTube-8M: https://research.google.com/youtube8m/, accessed on 18 July 2024.
